# Comparative insights into questions of lepidopteran wing pattern homology

**DOI:** 10.1186/1471-213X-6-52

**Published:** 2006-11-07

**Authors:** Antónia Monteiro, Gary Glaser, Steven Stockslager, Nelleke Glansdorp, Diane Ramos

**Affiliations:** 1 Department of Biological Sciences, University at Buffalo, 109 Cooke Hall, Buffalo, NY 14260, USA; 2 Institute of Biology, Leiden University, Kaiserstraat 63, P.O. Box 9516, 2313 RA Leiden, The Netherlands; 3 Department of Ecology and Evolutionary Biology, Yale University, 326A OML, 165 Prospect Street, New Haven, CT 06511, USA

## Abstract

**Background:**

Butterfly and moth eyespots can share a similar appearance, involving multiple concentric rings of colored scales, but usually occuring in non-homologous positions on the wing. Within the butterflies, on the other hand, spots that share the same homologous position may not share the concentric ring structure; and, in butterfly species that have eyespots with concentric rings, ectopic eyespots with a similar ring structure can be induced by means of a simple epidermal wound. The extent to which all these eyespots, natural or induced, share similar genes and developmental mechanisms is investigated here by means of protein in-situ localizations in selected butterfly and moth species. In addition to looking at some of the transcription factors previously identified as being involved in eyespot formation, we also tested the involvement of candidate genes from the Wingless and TGF-β signaling pathways as putative morphogens for eyespot development.

**Results:**

Saturniid moth and nymphalid butterfly eyespots with concentric rings of color express at least two transcription factors, Distal-less and Engrailed, in the center of the future pattern. Nymphalid eyespots centers also express the ligand Wingless and an activated signal transducer, a phosphorylated Smad protein, but neither these proteins nor the previous two proteins are found in pierid spot centers, which consist of a single patch of color. Both butterfly wing patterns, however, express a third transcription factor, Spalt, a portion of whose expression domain maps to the black scales on the adult wing. Wounding a nymphalid wing, on the other hand, leads to upregulation of *Distal-less, engrailed *and *spalt *in subsets of cells around the wounding site, mimicking concentric eyespot development.

**Conclusion:**

Wingless and TGF-β ligands are both candidate morphogens involved in nymphalid butterfly eyespot formation. These eyespots, as well as saturniid moth eyespots with concentric circles, share two genes that are associated with the differentiation of the signaling cells in nymphalid eyespots. This commonality suggests that they may be produced via the same developmental mechanism despite their non-homologous location. By contrast, pierid butterfly spots of a single color share some of the same genes but appear to be produced by a different mechanism. Eyespots with concentric rings may have co-opted a wound healing genetic network during their evolution.

## Background

The wings of butterflies and moths display a wealth of color patterns that provide excellent material for investigating the evolution of pattern formation in a simple, two-dimensional system. Pattern elements consisting of one or more concentric rings of colored scales, the eyespots, can occur at different positions in the wing in different lineages and also display different morphologies and, thus, make intriguing subjects for investigating questions of homology [1]. When eyespots appear in moth lineages they are usually found as a single element in each wing surface, straddling a cross vein in the center of the wing. These are called the discal-cell eyespots [2]. In members of the superfamilies Bombycoidea, Drepanoidea and Geometrodeia, which are closely related to the butterfly superfamily Papilionoidea, eyespots also appear along the border of the wing, in the space between two veins [3]. These border eyespots, common in several butterfly families, are part of one of the three anterior-posterior bands of pattern symmetry, the "distal symmetry system", as described for the Nymphalid Groundplan (NGP)[2]. Whereas many eyespots in moths and butterflies display a central pupil and several rings of concentric colors, some "eyespots" consist of patches of a single color. The extent to which discal-cell eyespots are homologous to border eyespots and to which single colored spots are homologous to eyespots with concentric rings remain largely unknown and is here the focus of our investigation.

Ideally, tests of homology should include not only comparisons at the level of the phenotype but also of the genes and developmental processes underlying that phenotype [4,5]. This type of homology is usually referred to as "process homology" [5]. The idea is that similar morphologies at non-homologous positions may be the result of homologous genes and developmental mechanisms that have been co-opted to novel locations, or alternatively, the result of disparate developmental processes that have converged on a similar morphology. In this study homology between structures will be examined at each of these levels. Below we briefly review what is known about eyespot development and introduce the candidate genes that will be used in our comparative study.

Research on eyespot developmental mechanisms has mostly focused on the border eyespots of nymphalid butterflies. Two and a half decades ago, Nijhout proposed that a group of signaling cells, the focus, organizes the differentiation of butterfly eyespot patterns by producing a long range diffusible morphogen that is interpreted in a threshold-like fashion by the surrounding epidermal cells [6]. When the focus is transplanted to a different location of the wing, an eyespot pattern differentiates in the surrounding host tissue [6,7]. Other subsequent models, where the focus acts as a "sink" for a broadly distributed morphogen [7-9], or where different concentric rings of cells act as new sources of morphogens through a cell relay system [10] have also been proposed for the border eyespots.

The sink model, in particular, is derived from the observation that eyespot mimics, containing rings of differently colored scales but no central white pupil, can differentiate on the wing following pupal epidermal wounding [7-9]. The wound was thus interpreted as a "sink" for a widely distributed morphogen. The effects of wounding in altering butterfly wing scale coloration have been investigated in different moth and butterfly species [7-9,11-14]. In all cases, there is a specific time period during pupal development when wounding of the wing epidermis will alter the fate of the color scales in an area surrounding the wound site. In most cases, the altered patch of cells is circular in shape and centered on the puncture site. In addition, punctures performed in different areas of the wing can result in ectopic eyespots of different sizes or different colors [9,11,12,15,16]. In *Bicyclus anynana *(Lepidoptera, Nymphalidae), ectopic eyespots consisting of a ring of gold scales, sometimes containing a black central disc, appear on the adult wing following pupal wing wounding [7]. The genes involved in the differentiation of these ectopic coloration patterns have not been investigated and, thus, it is unclear whether natural eyespots and ectopic eyespots are using the same genetic circuitry.

A series of genes have been associated with nymphalid eyespot development. In the larval stage, *Distal-less *(*Dll*), *Notch *(*N*), *engrailed *(*en*), *hedgehog *(*hh*), *cubitus interruptus *(*ci*), *patched *(*patch*), *spalt (sal) *(Craig Brunetti, personal communication) appear to be involved in the differentiation of the central signaling cells [17-19]. Later, in the early pupal stage, three of these early transcription factors are expressed again in a subset of epidermal cells, the scale-building cells, and appear to map to the adult eyespot color rings: Dll and Sal mapping to the black scales, and En to the gold ring of scales [20,21]. The identity of the signal that is produced in the focal cells that triggers the expression of these transcription factors is not yet known.

Here we investigate whether the Wingless (Wg) and Transforming Growth Factor-beta (TGF-β) signaling pathways are involved in differentiating the border eyespots, as the ligands in these pathways are known morphogens in other systems (reviewed in [22]). Using immunohistolocalization, we target the Wg ligand, and a phosphorylated form of a signal transducer from the TGF-β pathway, pSmad, as well as three of the previously described transcription factors Dll, En and Sal. We look at these proteins throughout the late larval and early pupal stages of the butterfly *B. anynana*. We also follow the expression of these proteins around wound sites in the pupal wing to determine the extent to which ectopic eyespots share developmental pathways with normal eyespots. Finally, we compare gene expression patterns in the discal-cell eyespots of the moth species *Antheraea polyphemus *and *Saturnia pavonia *(both Lepidoptera, Saturniidae) and in the uniformly-colored, non-pupilated, spots of the cabbage white butterfly *Pieris rapae*, from a more basal butterfly family (Lepidoptera, Pieridae), to determine to what extent they are sharing similar developmental pathways.

## Results

### *wg *and pSmad are both expressed in *B. anynana *border eyespots during the early pupal wing stage

**Figure 1:**
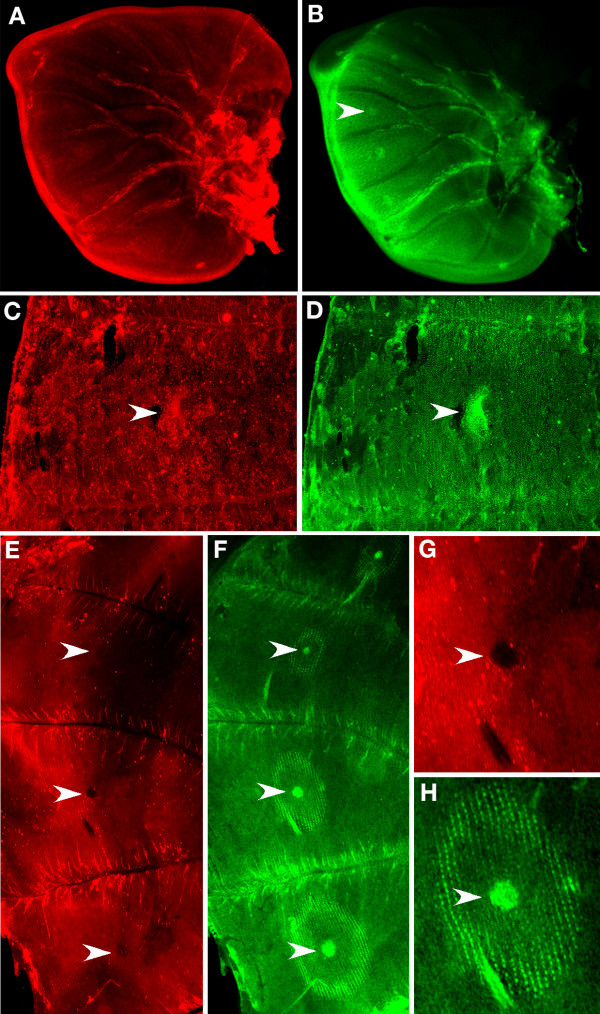
***wingless *expression in *Bicyclus anynana *larval and pupal wings**. (A, B) Joint visualization of Wg (red) and En (green) in late 5th instar larval wing discs (50×); (A) Wg is present in the distal margin but not in the future eyespot centers in late 5th instar larval wing discs; (B) En is present in the future eyespot centers and in the posterior wing compartment; (C, D) Wg is present in the future eyespot centers at 12 h after pupation and its expression if coincident with that of En in the focus (large posterior forewing eyespot depicted) (100×); (E, F) Joint visualization of Wg and En at 16.5 h after pupation, while Wg levels (E) drop in the future eyespot centers, En (F) is still present in the future eyespot centers (50×); (G, H) Enlargement of 4^th ^hindwing eyespot (200×). Arrowheads point to eyespot centers in all panels.

**Figure 2:**
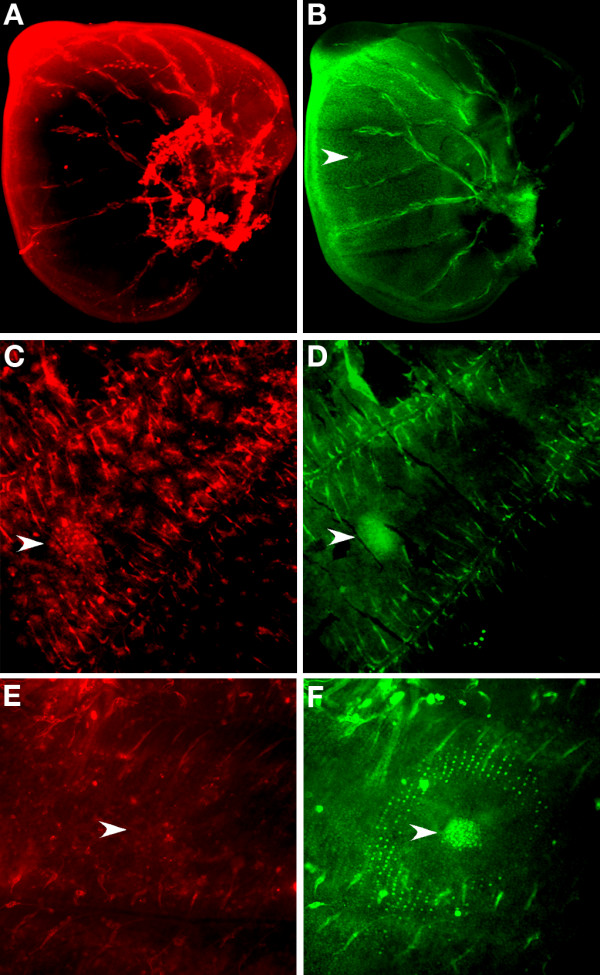
**pSmad expression in *Bicyclus anynana *larval and pupal wings**. (A, B) Joint visualization of pSmad (red) and En (green) in late 5th instar larval wing discs (50×); (A) pSmad is present along the margin but is absent in the future eyespot centers (arrowhead) where En (B) is visible; (C) pSmad and En (D) are present in the future eyespot centers, at 12 h after pupation. Note the patchiness of the pSamad staining outside of the focal area, perhaps indicating centers of epidermal cell growth (100×); (E) pSmad is no longer visible in the eyespot center (arrowhead) at 18 h after pupation, whereas *en *expression (F) extends to a ring pattern of scale-building cells surrounding the focus (200×).

Stainings for Wg and pSmad, performed during the late fifth larval instar, showed expression along the wing margin but no expression in the focal cells (Fig. 1A &2A). Stainings performed in the early pupal wing, however, show that Wg (Fig. 1C) and pSmad (Fig. 2C) are both present in the eyespot field. At later pupal stages, when the ring of *en*-expressing scale-building cells is visible around the eyespot focus (Fig. 1F &2F), Wg and pSmad are no longer visible in the eyespot field (Fig. 1E &2E).

**Figure 3:**
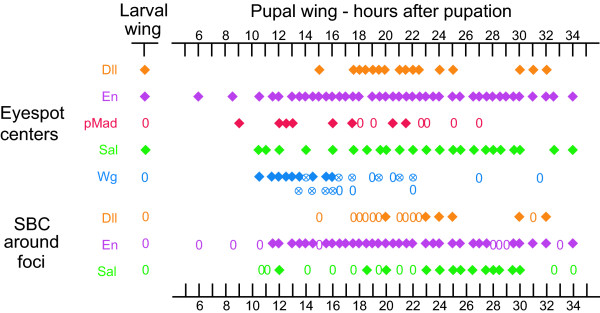
**Timing of visualizations of Dll, En, pSmad, Sal, and Wg in eyespot centers and scale-building cells (SBC) in larval and pupal wing discs**. Each symbol along a row represents data for a unique individual, whereas symbols across rows may represent the same individual. Diamonds represent presence of the protein, zeroes represent absence of the protein, and circles with crosses represent absence of the protein below background levels. The second Wg row represents less common patterns to those observed and depicted in the first row, for the same time period.

The timing of *wg *expression varied slightly from individual to individual with expression first visualized in the eyespot centers at 10.5 h after pupation (earlier wing dissections were difficult to perform) and present until 16 h after pupation (Fig. 3). During this period there were dynamic patterns of *wg *expression. In particular, the high levels of expression seen in the focus early after pupation in both fore and hindwings (see Fig. 1C for forewing expression) decreased relative to levels of background staining in the hindwing (Fig. 1E,G), slightly later in development. These lower levels of Wg were not observed in the forewing, however. After 22 h post pupation, there was no longer any obvious pattern of *wg *expression in both fore and hindwings (Fig. 3).

**Figure 4:**
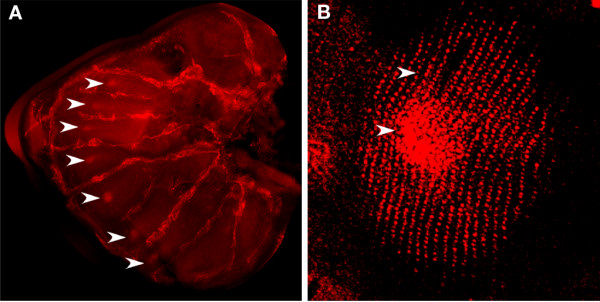
***spalt *expression in *Bicyclus anynana *larval and pupal wings**. (A) *sal *is expressed in the future eyespot centers (arrowheads), in the late 5th instar larval hindwing discs (50×); (B) *sal *expression extends to the scale-building cells in a larger circular pattern around the densely packed cells of the eyespot focus, at 19 h after pupation (arrowheads point to focus and to scale-building cells) (200×).

pSmad expression varied slightly from individual to individual with expression first visualized in the eyespot centers at 9 h after pupation and present until 18 to 21.5 h after pupation, depending on the individual (Fig. 2C &3). From 22 h onwards, pSmad was no longer detected in the eyespot centers (Fig. 2E). At 9 h after pupation, pSmad was also expressed in small cell clusters spread throughout the wing epidermis, possibly indicating centers of epidermal wing growth (Fig 2C).*Dll, en *and *sal *were all expressed in the eyespot centers during the late 5th larval instar (Figs. 3, 4A, Dll staining not shown), but only began to be expressed in the surrounding scale-building cells later after pupation (at 12 h to 20 h), possibly responding directly to levels of one or both of the putative morphogens proposed here.

### Only *sal *and pSmad are expressed in the region of *P. rapae *border spots

**Figure 5:**
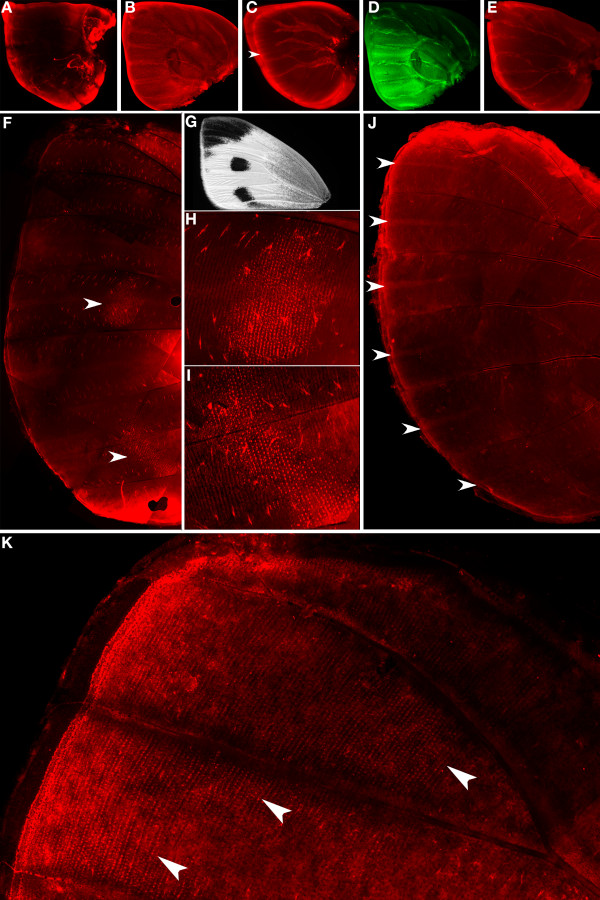
**Expression of pSmad, *wg, Dll, en*, and *sal *in *Pieris rapae *larval and pupal wings**. (A-E) Late 5th instar larval discs (50×); (A) pSma*d *is present in the distal margin; (B) Wg is also present in the distal margin and throughout the wing; (C) *Dll *is expressed in the distal margin and in intervenous stripes between wing veins (arrowhead); (D) *en *is expressed in the posterior compartment as well as along two intervenous stripes in the wing compartments that later carry the black spots; (E) *sal *is expressed in the distal margin; (F) *sal is *expressed in scale-building cells in the future black spots at 25.5 h after pupation (arrowheads) (100×); (G) *Pieris rapae *adult forewing showing two black spots and a black wing tip; (H) and (I) Enlargement of patches of *sal *expression from (F) (100×); (J) *Dll *is still expressed in intervenous stripes leading from the distal margin at 25.5 h after pupation (arrowheads) (100×). (K) Sal is present in scale-building cells at the distal tip of the wing at 26 h after pupation (arrowheads mark boundary of *sal *expression) (100×).

pSmad and *wg *were expressed along the wing margin of *P. rapae *(Fig. 5A,B) but unlike *B. anynana, P. rapae *larval wings do not appear to have a differentiated cluster of cells expressing *Dll, en*, and *sal *at the center of the future black spots of pigmentation (Fig. 5C–E, Fig. 4A). This was concluded after more than 50 forewings were analyzed, sampling from the last larval, crawler, and pre-pupal stages. Note, however, that En is present along two intervenous stripes in the wing sectors that later carry the black spots. In the pupal wings of these butterflies there was also no visible cluster of cells expressing pSmad or *wg *in the future spot centers shortly after pupation. Although we performed pupal stainings with the collection of antibodies targeting the proteins mentioned above, Sal was the only protein investigated that mapped to the adult spots (5F-I), whereas Dll was expressed in the intervenous stripes (Fig. 5J). Sal was visible in scale-building cells from as early as 13.5 h after pupation till 27 h after pupation (subsequent developmental times were not investigated). Sal was also present in scale-building cells along the black tip region of the wing during the same time periods investigated (Fig. 5K).

### Gene expression after pupal wing wounding

**Figure 6:**
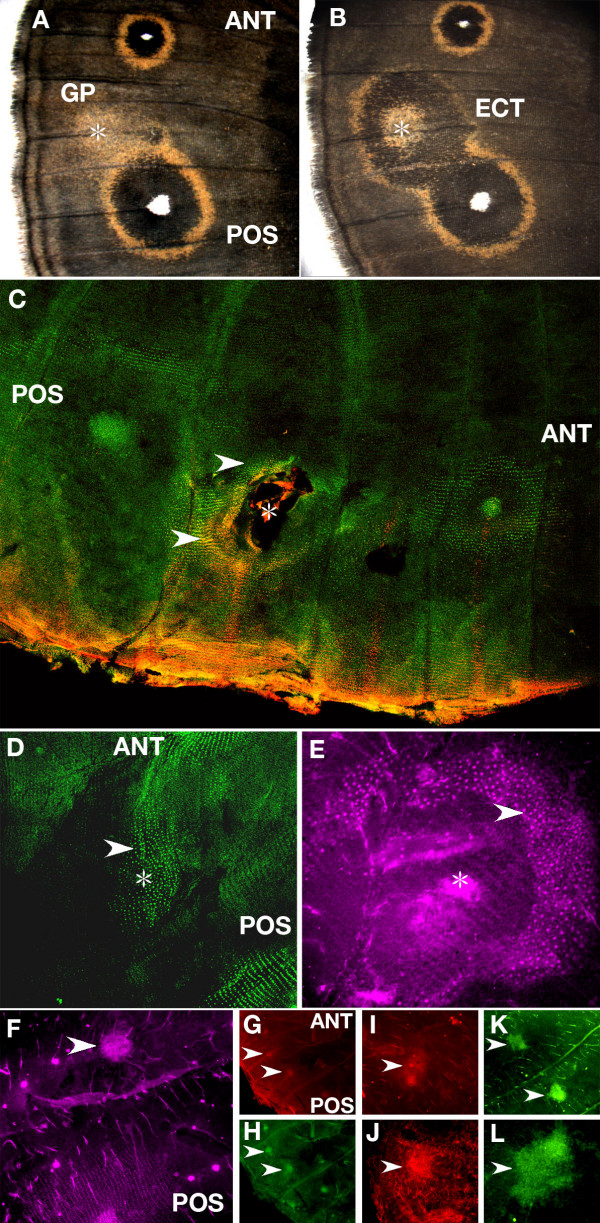
**Gene expression after epidermal wounding in *Bicyclus anynana***. (A) Gold patches (GP) of scales are produced in some individuals around a wound site (star symbol) between the anterior (ANT) and posterior eyespots (POS), whereas (B) ectopic eyespots (ECT) containing both black scales and gold scales differentiate in other individuals; (C) Dll (red) and En (green) are present in scale-building cells around a wound site (yellow represents co-expression). Epidermis was wounded at 12 h after pupation (W = 12 h) followed by tissue fixation and protein visualization at 25 h after pupation (V = 25 h) (50×); (D) *en *is expressed in a patch of scale-building cells (arrowhead) around wound site (star symbol) (W = 9 h, V = 24 h) (100×); (E) *sal *is expressed in scale-building cells (arrowhead) around a wound site (approximate location shown by star symbol) (W = 13.5 h, V = 28.5 h) (200×). The absence of a continuous epidermis in the center of the wound site in (C) and (E) is the result of damage during the process of wing detachment from the overlying cuticle due to the presence of a wound scab; (F-L) Antibodies bind to the center of a wound (shown in all panels by arrowheads) in a non-specific fashion; (F) anti-Sal antibody binds to a cluster of non-differentiated cells (W = 11.5 h, V = 24 h) (100×); (G) Anti-Dll (W = 9 h, V = 24 h) (50×); (H) Anti-En (W = 9 h, V = 24 h) (50×). Note the two flanking eyespot foci on this wing (G, H) expressing *Dll *and *en*; (I) Anti-Wg (W = 9 h, V = 24 h) (100×); (J) Anti-pSmad (W = 9 h, V = 24 h) (100×); (K) Control staining using only an anti-mouse secondary antibody (and no primary antibody) also showing expression at the center of two wound sites (W = 9 h, V = 24 h) at 50X, and at 200X (L).

**Figure 7:**
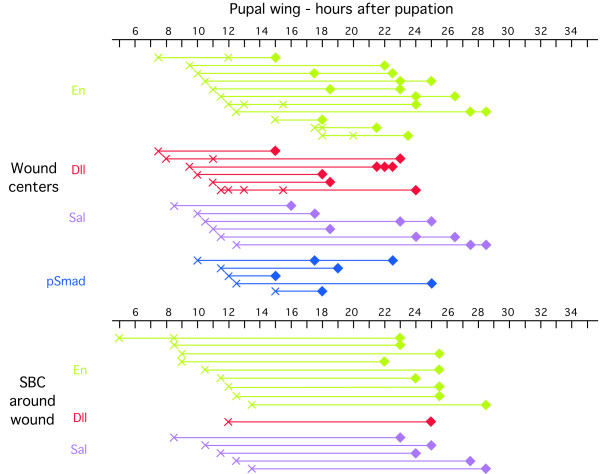
**Timing of visualizations of antibodies targeting En, Dll, and Sal at wound centers and in scale-building cells surrounding wound centers after epidermal wounding**. The age of the pupa when wounding was performed (cross symbol) is connected by an horizontal line to the age of the same pupa at the time the wing was extracted and fixed (diamond symbol), and stained with a particular antibody, Only wings where we observed some staining pattern are depicted in this figure. Data was condensed in the following way: more than one cross present on the same line represents observations in different individuals that were wounded at different times but whose wings were fixed at the same time after pupation. More than one diamond in the same line represent observations in different individuals that pupated at the same time and were fixed at different times. As explained in the text, the results for the wound centers (top part of the figure) may not represent real gene expression patterns, as non-specific antibodies were also shown to bind at these sites.

Wounding the pupal wing led to the production of gold scales or ectopic eyespots around the site of damage in the adult wing of *B. anynana *(Fig. 6A,B). Wounding also led to the up-regulation of *Dll, en*, and *sal *in a subset of epidermal cells, the scale-building cells, several hours (at least 12.5 h) after wounding (Fig. 6C–E; Fig. 7). From the cell-specific pattern of expression we interpret these results as real patterns of up-regulation of these genes in these cells. In all of the trials we observed either the expression of *en *alone in the scale-building cells, or both *Dll+en *or *sal+en *gene combinations expressed simultaneously in the same cells. This indicates that cells that are going to produce a single pigment later in development may have an earlier co-expression of more than one of the "color" transcription factors before putative interactions between them exclude one of the transcription factors from the same cell [21]. These specific combinations of transcription factors are not found in naturally occurring eyespots of *B. anynana *but are found in other butterfly species [21].

In many wings, however, there were no patterns of target gene expression in association with scale-building cells. These wings were normally operated and observed before 12 h had elapsed from the time of the operation. Instead there appeared to be a cluster of non-differentiated cells, at the site of wound closure that were visibly targeted by antibodies (Fig. 6F–H). This type of expression, however, was non-specific because control stainings (only using mouse secondary antibodies) showed the same pattern (Fig. 6K,L). Attempts to detect whether cells surrounding the wound site were producing pSmad and/or Wg, were fraught with the same problem probably due to some stickiness of the damaged tissue (Fig. 6I,J), indicating that our immunohistolocalization method is not optimized to detect these early signals in damaged butterfly epidermal tissue.

### *Dll *and *en *are expressed in discal-cell eyespots

**Figure 8:**
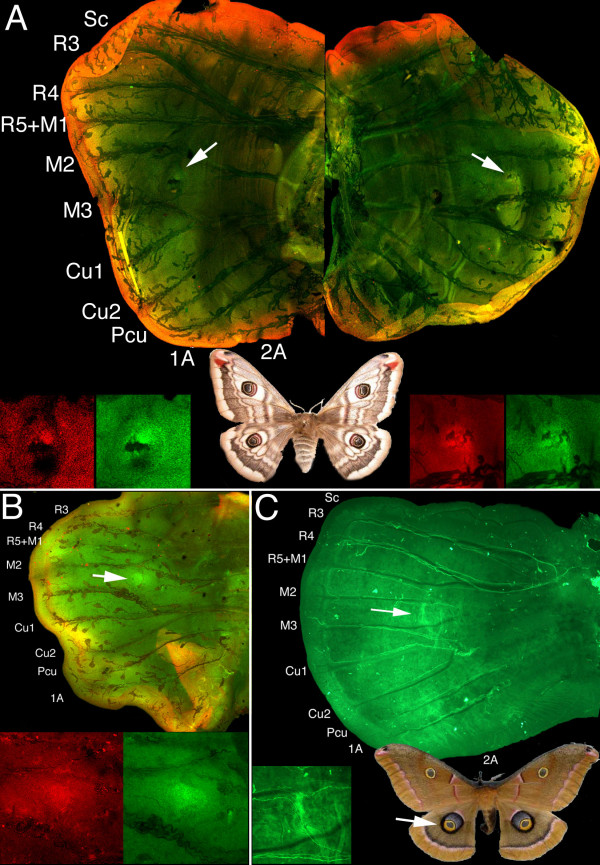
**Expression of Dll and En in two saturniid species in the late larval wings, two to three days after the start of cocoon spinning**. (A) Left and right forewings of *Saturnia pavonia*, showing Dll expressed in the margin (red) and also in future discal-eyespot centers (arrows and insets), together with En (green insets), two days after the beginning of cocoon spinning. The black marking next to the Dll- and En-expressing cells of the left wing appears to be a piece of debris, whereas the darker spots overlying these same cells on the right wing appear to be small pieces of trachea. (B) Hindwing of *Saturnia pavonia *showing an enlarged focus expressing Dll and En at the future discal-eyespot center. Note the absence of a cross vein at this stage. The area showing double staining in the bottom right sector of the wing represents a fold of the wing margin over part of the wing. (C) Hindwing of *Antheraea polyphemus *three days after beginning of cocoon spinning showing En present in a line marking the elongated central axis of the future discal-cell eyespot. In the pupal stages of both saturniids a cross vein will appear between M2 and M3, presumably at the position where En and Dll are expressed (see [42] for venation notation). All panels at 50× and insets at 100×.

Anti-En, and anti-Dll and-En co-stainings performed in two saturniid moths, *A. polyphemus *and *S. pavonia*, respectively, showed that *Dll *is expressed along the margin (Fig. 8A) and that both Dll and En are present in the center of the future discal eyespots during the late larval stage, just before pupation, but 2–3 days after the larva starts spinning its cocoon (Fig. 8). The focal expression of these genes seems to appear before any cross-vein is visible at this position (see insets on Fig. 8A–C).

## Discussion

### The candidate morphogens *wingless *and TGF-β ligands

One of the central aims of these experiments was to identify additional proteins for comparative work including putative morphogens involved in focal signaling and eyespot differentiation, as originally proposed by Nijhout [6]. Given that we knew the identity of transcription factors that are possibly responding directly to morphogen levels produced in the focus [20,21], we asked whether up-stream regulators of these transcription factors were known in other systems. In *Drosophila*, Wg and the TGF-β ligand Dpp are known to activate *Dll *and *sal*, respectively, in a concentration dependent fashion [23-25]. For instance, during wing and leg development in *Drosophila*, *Dll *is responding directly to levels of Wg produced along the wing margin [23] and to levels of both Wg and Dpp produced in the imaginal leg disc [26]. *sal*, on the other hand, responds directly to levels of Dpp during anterior-posterior axial patterning in the *Drosophila *wing [27]. These ligands are known to form concentration gradients around the producing cells and to provide positional information to surrounding cells as to their proximity to the cells releasing the signal (.reviewed in [28]). Also, these interactions have been shown to be the result of a morphogen gradient system and not a cell-cell relay system.

Until functional data is collected for Wg and TGF-β family members, these ligands remain excellent candidates for association with focal signaling and eyespot differentiation due to their temporal and spatial localization. The presence of Wg and pSmad along the wing margin and their absence from the eyespot foci during the larval stage is in agreement with previous reports for the expression of *wg *and *decapentaplegic *(*dpp*), a ligand from the TGF-β family, in the nymphalid butterfly *Precis coenia *using *in situ *hybridizations [18]. Both Wg and pSmad, however, were present in the *B. anynana *eyespot centers from the early pupa until around 16–22 h after pupation, a critical developmental period when pupil transplants and ablation experiments were originally shown to affect the eyespot phenotype [7]. The genes were then down-regulated after this point. In our stainings we observed that both pSmad and Wg appear to be restricted to the area of the focus. Because biologically active levels of these proteins may be below the limits of antibody detection, these stainings should perhaps be viewed as indicating where protein concentrations are highest.

### Gene expression at wound sites

The expression of *Dll, en*, and *sal *was only observed in scale-building cells around the wound site from 12 h onwards after the wounding operation. These patterns of gene expression mimic the temporal and spatial patterns of genes expressed around the natural eyespot centers where Dll, En and Sal only appear in scale-building cells at least 12 h after pupation (Fig. 3). This temporal delay between signaling from the focus (or putatively from a wound) and expression of the genes in scale-building cells could represent the time that it takes morphogen levels to build up before response genes can be activated and proteins detected. It remains to be determined whether *Dll, en, and sal *could have been up-regulated by any of the two putative morphogens proposed above, produced at a wound site. Other studies have linked Dpp and Wg to the regeneration of damaged tissue in vertebrates [29-31] but our antibody stainings do not provide conclusive evidence for Wg and pSmad being up-regulated around wound sites immediately after damage because dying or non-differentiated cells at these sites appear to bind non-specifically to antibodies. The expression of *Dll, sal and en *in the sub-set of scale-building cells following wounding is likely to represent a derived feature of butterflies with eyespots rather than a functional aspect of wound repair *per se*. This proposition, however, can only be addressed by comparative work in wound repair in other parts of the butterfly body or in other organisms. Also, future work targeting mRNAs, instead of proteins, may be able to determine whether TGF-β or Wg mRNAs are indeed produced at the wound sites.

Since both Wg and TGF-β have known roles in wound healing in other systems, their involvement in eyespot development within the Lepidoptera leads us to speculate that perhaps eyespot evolution resulted from the genetic co-option of genes from a wound repair genetic circuitry in specific regions of the wing, followed or proceeded by co-option of pigmentation pathways to the end of that circuit. It has been proposed before that eyespots represent "flat legs" on a wing [18], having resulted from the co-option of the leg developmental program involving the *Dll *gene [32]. Our results suggest an alternative to this scenario where eyespots, as well as legs and other appendages, are derived from a putatively more ancestral circuit involved in tissue repair [33]. Appendages would have co-opted and modified the wound repair program in order to grow tissue outward, whereas butterflies used it to color-pattern the wing.

### Eyespot development across species

From our comparative analyses of moth and butterfly eyespots, it appears that border eyespots of nymphalid butterflies and discal-cell eyespots of Saturnid moths share at least two genes that are associated with the differentiation of the group of central signaling cells, *Dll *and *en*, and may, therefore, share similar developmental mechanisms. Due to the small colony of animals reared we did not collect sufficient additional data to test for the expression of the remaining genes and were unsuccessful at collecting data for the pupal stage. Future gene expression studies as well as experiments involving discal-cell focal grafts or damage experiments should shed additional light on the underlying developmental mechanisms for these eyespots.

In contrast to the border and discal-cell eyespots, the uniformly colored non-pupilated spots of *P. rapae *seem to be produced through a different developmental mechanism that does not involve signaling from the center. The spots in *P. rapae *do not appear to have a group of differentiated cells at their center, i.e., the spots do not express any of the focal markers (Dll, En and Sal) in the larval wing stage, and they do not have pSmad or *wg *expression at their centers during the early pupal stage. These spots, however, share the expression of the transcription factor, Sal, which is associated in both *B. anynana *and *P. rapae *with the area of black scales in the adult wing. In addition, in the late larval wings of both species, pSmad is first visible along the wing margin (Fig. 5A). The known activation of *sal *at particular threshold levels of pSmad in *Drosophila *[27] is suggestive that these two patterns may be causal, i.e., a putative gradient of a TGF-β protein could be established at the wing margin in late larval wing development, leading to the putative activation of a band of *sal *expressing cells at some distance from the margin in the early pupa.

The restriction of Sal in the *Pieris *wing to only two spots along a putative continuous anterior-posterior gradient of TGB-β signaling may be a result of one of three proposed mechanisms. First, a series of transcription factors homologous to those patterning the *Drosophila *wing along the anterior-posterior axis and positioning the veins [17,34] may be subdividing the butterfly wing into a series of genetic domains [35]. These genes could allow for certain areas of the wing to express *sal *while repressing it in other areas. Second, the expression of *en *in the two intervenous regions that will later carry the spots could be involved in restricting *sal *expression to those sectors of the wing, either by activating it directly, or by repressing a general *sal *repressor distributed more broadly across the wing, and allowing *sal *to be expressed in those areas. Thirdly, if *sal *has a modular cis-regulatory region where each module is responsible for the gene's expression in a different anterior-posterior section of the wing, pSmad, in combination with other regional regulators (for instance the transcription factors involved in vein positioning) may be simultaneously needed to activate one or more *sal *cis-regulatory modules along a continuous stripe on the wing.

### Homology of eyespot systems

**Figure 9:**
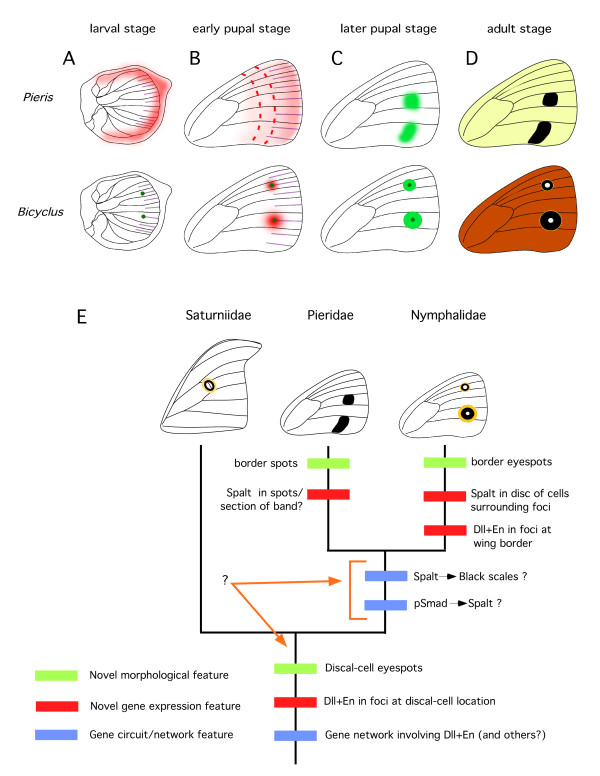
**Model for nymphalid eyespot and pierid spot development, and evolutionary hypothesis of spot/eyespot evolution**. (A) During the late larval stage, *Dll *and *Notch *are expressed in several intervenous stripes (purple) midway between the wing veins in both *B. anynana *and *P. rapae *butterflies, but only a sub-set of wing cells express these and several other genes in enlarged foci at the end of those stripes, including *sal *(dark green; forewing represented here where only two foci differentiate). PSmad (red) is also present along the wing margin; B) In the early stages of pupal development, pSmad (red) is expressed in the future eyespot centers of *B. anynana*, but not in the spot centers of *P. rapae*. A gradient of this protein, however, may be present along the proximal-distal axis of the wing, being established from the marginal expression of TGF-β ligands in the late larval wings. This gradient could display a similar range of concentrations along bands spanning the anterior-posterior axis of the wing (red dashed lines); C) Later in pupal development while in *B. anynana sal *(light green) responds to pSmad levels generated from focal signaling, in *P. rapae*, *sal *is responding to particular pSmad levels expressed along the anterior-posterior axis (see text for further explanation of spot pattern). (D) The expression of *sal *determines where black scales will develop on the adult wing in both species. (E). Phylogenetic tree depicting a member from each lepidopteran family studied. The appearance of novel (non-homologous) features are depicted on the tree. These features can represent a novel phenotype, a novel gene expression pattern, or a novel gene circuit (see text for description). Note that additional information was used to map the origin of the discal-cell eyespots at the base of the tree, rather than in the branch representing the saturniid lineage, which would have been more parsimonious (see text).

Despite previous morphological examinations having proposed that the elaborate border eyespots found in many nymphalids are not homologous to the simple spots found in other butterfly lineages such as in the pierids [2,36], an in-depth examination of the underlying developmental basis of these patterns had not been attempted. Our data suggest that the two pattern elements have evolved independently by deploying separate developmental mechanisms, but happen to utilize some of the same genes, e.g. *sal*, and perhaps two of the same pre-existing and thus, homologous, gene circuits, e.g. pSmad being upstream of *sal *activation, and sal being upstream of black scale differentiation. These two circuits may also be ancestral to the butterfly-moth lineage split but our current lack of data for the expression of these genes in the saturniids does not allow us to examine this proposition. In any event, because the mechanism of how *sal *is eventually activated in the pupal stage in *Pieris *and *Bicyclus *scale-building cells is proposed to be different for each system (Fig. 9B,C): either due to a threshold response to a gradient of a TGF-β ligand diffusing from a central group of signaling cells, or from a gradient expressed along the entire wing, that turns on *sal *in selective quadrants along the anterior-posterior axis, the two spots should not be considered homologous at the level of developmental mechanisms.

In the case of the saturniid and nymphalid eyespots with concentric rings it appears that both these patterns have a differentiated focus that expresses similar transcription factors (e.g., En and Dll), that could lead to a similar focus signaling mechanism, but whether the systems have independently evolved in their non-homologous positions by the co-option of an ancestral focus gene circuit into a novel position (as represented in Fig. 9E), or retained similar mechanisms throughout Lepidoptera evolution that merely shifted in location is still unclear. Only a broad sampling across several more butterfly and moth lineages, including those that appear to have both a discal-cell eyespot and border eyespots, will be able to shed light on this issue. In any event, and with the current data, we propose that these two spots are "process homologous" at the levels of genes and developmental mechanism.

Our mapping of the co-option of a *Dll *and *en *gene circuit and the appearance of discal-cell eyespots at the base of the tree (Fig. 9E), rather than within the saturniid lineage, is not the most parsimonious reconstruction for the data analyzed here. We chose this location for two reasons. First, many other moth lineages, as well as pierids (e.g. *Colias sp*.), display these discal-cell eyespots, making their appearance at the base of the tree more parsimonious. Second, it was previously shown that *Dll *is also expressed at a discal-cell location in a nymphalid butterfly [37]. Denser species sampling, however, is also needed to conclusively resolve this issue.

The genetic circuitry involved in nymphalid eyespot production, with its putative signaling ligands (Wg and TGF-βs) and several response genes (*Dll, en *and *sal*) may, at least in part, have been co-opted from the wound repair genetic circuitry, as the two developmental processes share many temporal and genetic similarities. Future work on wound healing across a broader range of species should help verify this hypothesis.

## Conclusion

We propose that both Wingless and TGF-β are involved in signaling from the future eyespot centers in nymphalid butterflies and directly or indirectly activating the expression of response genes such as *Dll, sal*, and *en *in concentric rings of cells surrounding the focal cells. We also propose that saturniid moth and nymphalid butterfly eyespots are sharing a similar developmental mechanism despite their non-homologous position on the wing, whereas pierid and nymphalid border spots appear to be produced through non-homologous developmental processes, despite sharing a homologous position and some of the same genes and putative gene circuits. The genetic basis for the development of all eyespots with concentric circles may have been co-opted from a wound healing genetic mechanism.

## Methods

*B. anynana *were reared at 27°C and 80% humidity in a 12:12 light:dark cycle. *P. rapae *were reared at 25°C in a chamber with no humidity control with a 16:8 light:dark cycle. *S. pavonia *were reared in sleeve cages outdoors, and *A. polyphemus *were reared at room temperature indoors (20°C). Pupation times for *P. rapae *and *B. anynana *were scored by means of time-lapse photography with a Kodak 290 digital camera, inside the 27°C and 80% humidity environmental chamber. The time-lapse was set to take pictures every 30 minutes with a time stamp on each photo. We dissected roughly 200 pairs of pupal wings and 100 larval wings from *B. anynana*, 60 pairs of pupal forewings and 50 larval forewings from *Pieris*, 12 larval wings from *S. pavonia *and 80 larval wings from *A. polyphemus*. Larval and pupal wings were fixed and stained following the protocol in reference [21]. We used a double-staining procedure for most wings with a mouse anti-En (4F11, 1:5 dilution [38]) for all preparations combined with either rabbit anti-Dll (1:200 dilution [39]), rabbit anti-Phosphorylated-SMAD1 (Ps1 [40]) named here pSmad at 1:150 dilution), rabbit anti-Sal (1:500 dilution [41]) and goat anti-Wg (1:200 dilution, Santa Cruz Biotechnology #SC-6280). The pSmad antibody specifically recognizes the phosphorylated form of the Smad protein and has been successfully used in vertebrates and in *Drosophila*., thus serving as an indicator of TGF-β activity on the butterfly wing. The Wg polyclonal antibody was developed against a conserved epitope in the region between amino acids 25–75 of the Human Wnt-1 protein (Swiss-Prot #P04628, GenBank #X03072). The epitope is proprietary and therefore only a range of amino acids was provided for where the epitope is located. In this 50 a.a. region there is 35.3% identity among amino acids when compared to *Bombyx mori*, the closest species with a fully sequenced *wg *gene. Within the same region there is a smaller group of 12 amino acids (#64–75) that have 75% identity with the *Bombyx *sequence. We used Alexa Fluor 488 anti-mouse with either Alexa Fluor 594 anti-goat or Texas-Red anti-rabbit secondary antibodies (Molecular Probes catalogue numbers A-11001, A-11058 and T-2767). Wings were mounted on glass slides in Slow Fade medium (Molecular Probes catalogue #S-7461), covered with a glass coverslip, sealed with clear nail polish, and stored at -20°C until observation under FITC and Texas Red filters on a *Leica *DMIRE II microscope at 50X, 100X and 200X magnifications.

In order to induce a wound and healing response we pierced individual pupal wings at one or two of three different sites with a fine tungsten needle (World Precision Instruments, catalogue #501317). Operations were performed between 5 h and 20 h after pupation (AP), covering the time period where most ectopic eyespots are produced, between 12 h and 18 h AP [9]. Pupae were punctured under a dissection microscope and the puncture depth ranged between 2 and 3 millimeters. Wings were dissected at a variable period after the operation and stained according to the protocols above.

Images shown represent the best specimens for each treatment but similar patterns were observed in at least 3 other specimens of each species, and often many more. Control stainings were also performed for all the developmental stages of *Pieris *and *Bicyclus *using combinations of secondary antibodies only. These stainings, apart from the ones mentioned explicitly in the text in the context of the wound healing experiments, yielded very dark preparations with no particular patterns of fluorescence.

## Authors' contributions

AM conceived the study, collected the data for the moths, part of the data for *P. rapae *and the wounding experiments, and drafted the final manuscript. GG collected the Wingless data, part of the pSmad data for *B. anynana *and *P. rapae*, and put together the first draft of the manuscript. SS collected part of the data for pSmad in *B. anynana *and, together with NG, collected part of the data for the wound healing experiments. DR helped draft the manuscript. All authors read and approved the final manuscript.
